# Crystal structure of ethyl 6-chloro­methyl-2-oxo-4-(2,3,4-tri­meth­oxy­phen­yl)-1,2,3,4-tetra­hydro­pyrimidine-5-carboxyl­ate

**DOI:** 10.1107/S2056989015011688

**Published:** 2015-06-20

**Authors:** M. Suresh, M. Syed Ali Padusha, J. Josephine Novina, G. Vasuki, Vijayan Viswanathan, Devadasan Velmurugan

**Affiliations:** a PG & Research Department of Chemistry, Jamal Mohamed College (Autonomous), Tiruchirappalli-20, India; b Department of Physics, Idhaya College for Women, Kumbakonam-1, India; c Department of Physics, Kunthavai Naachiar Government Arts College (W) (Autonomous), Thanjavur-7, India; d Centre of Advanced Study in Crystallography and Biophysics, University of Madras, Guindy Campus, Chennai-25, India

**Keywords:** crystal structure, conformation, di­hydro­pyrimidine, ring motif, C—H⋯π inter­actions

## Abstract

In the title compound, C_17_H_21_ClN_2_O_6_, the di­hydro­pyrimidine ring adopts a flattened envelope conformation, with the *sp* ^3^-hybridized C atom forming the flap. The dihedral angle between the least-squares planes of the benzene and di­hydro­pyrimidine rings is 88.09 (6)°. An intra­molecular C—H⋯O hydrogen bond generates an *S*(6) ring. In the crystal, mol­ecules are linked *via* pairs of N—H⋯O hydrogen bonds, forming inversion dimers with an *R*_2_^2^(8) ring motif, and the dimers are linked *via* further pairs of N—H⋯O hydrogen bonds, forming *R*_2_^2^(14) rings and chains of mol­ecules along [111]. Pairs of inversion-related chains are linked *via* weak C—H⋯π inter­actions.

## Abstract 2

The di­hydro­pyrimidine ring in the title ester adopts a flattened envelope conformation. An intra­molecular C—H⋯O hydrogen bond generates an *S*(6) ring. Mol­ecules are linked *via* pairs of N—H⋯O hydrogen bonds, forming inversion dimers.

## Chemical context   

Pyrimidine derivatives have been investigated extensively due to their great biological significance and as the main constituent of nucleic acids. Pyrimidines and their derivatives are considered to be important for drugs and agricultural chemicals. They are also found to exhibit remarkable pharmacological activities such as anti-cancer, anti-tumor, anti-inflammatory and anti­fungal *etc* and are used widely as agrochemicals, pharmaceuticals, dyes, organic additives in electroplating of steel and in the polymerization process (Sharma *et al.*, 2014 ▸; Vaisalini *et al.*, 2012 ▸). Di­hydro­pyrimidino­nes, the product of the Biginelli reaction, are also widely used in the pharmaceutical industry as calcium channel blockers and alpha-1 antagonists (Beena & Akelesh, 2012 ▸). Moreover, some bioactive alkaloids such as batzelladine B, containing the di­hydro­pyrimidine unit, which has been isolated from marine sources, show anti-HIV activity (Asghari *et al.*, 2011 ▸). Our inter­est in the preparation of pharmaco­logically active compounds led us to synthesize the title compound (I) and we report its crystal structure herein.

## Structural commentary   

**Figure 1:**
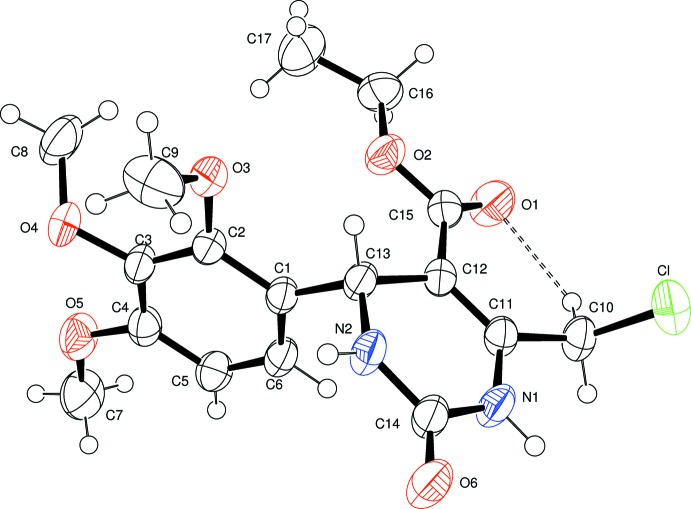
The mol­ecular structure of the title compound, with displacement ellipsoids drawn at the 50% probability level. The dashed line indicates the intra­molecular C10—H10*A*⋯O1 hydrogen bond.

The mol­ecular structure of (I) is shown in Fig. 1 ▸. The di­hydro­pyrimidine ring adopts a flattened envelope conformation. Atoms N1/N2/C11/C12/C14 are essentially planar with a maximum deviation of 0.0305 (17) Å for C11 while atom C13 is displaced by 0.1311 (17) Å from this plane, forming the flap. The puckering parameters are *q*2 = 0.0935, *q*3 = −0.0317, *Q* = 0.0987 Å, Θ = 108.7 and Φ = 22.9°. The benzene ring is almost perpendicular to the least-squares plane of the six-membered tetra­hydro­pyrimidine ring, making a dihedral angle of 88.09 (6)°.
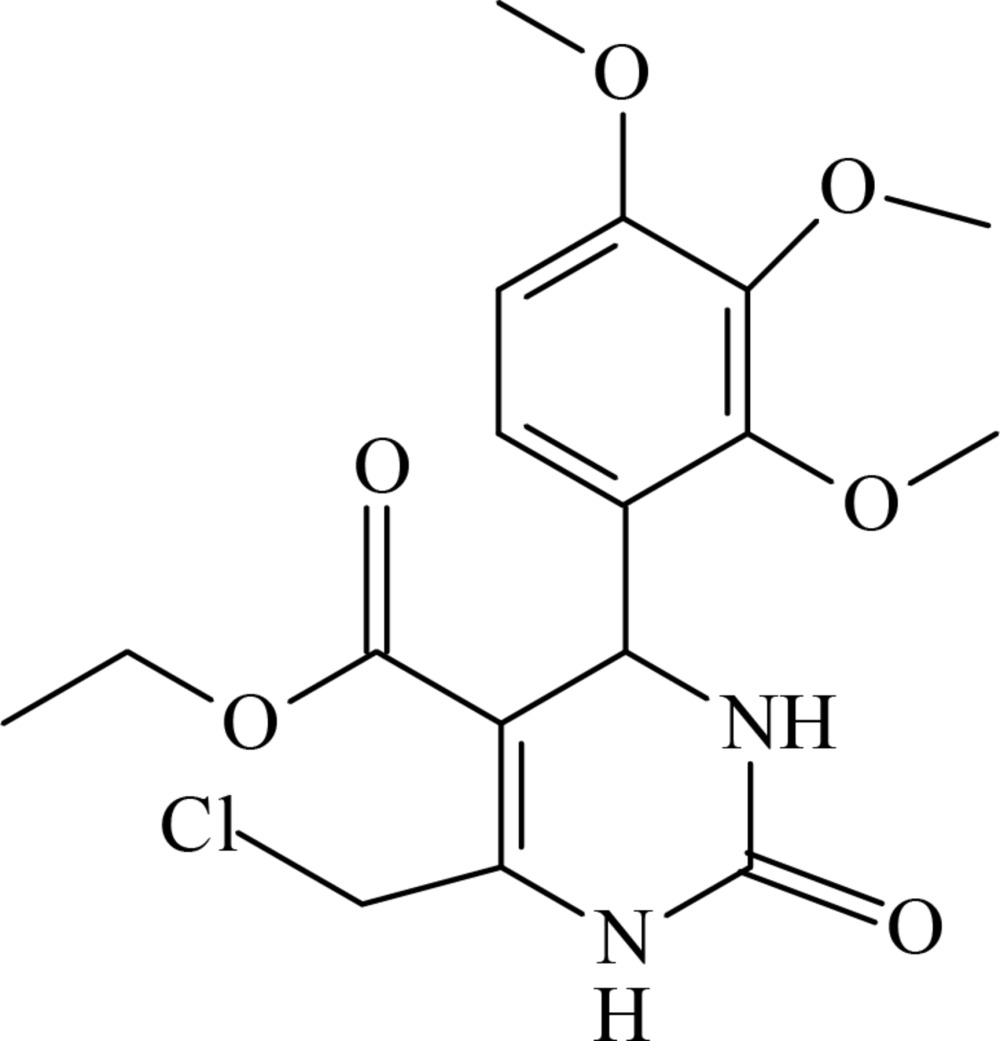


In comparison, this dihedral angle in the structure of ethyl 6-eth­oxy­carbonyl­methyl-4-(2-hy­droxy­phen­yl)-2-oxo-1,2,3,4-tetra­hydro­pyrimidine-5-carboxyl­ate, (II), is 87.7 (2)° (Kettmann *et al.*, 2008 ▸), in ethyl-6-(chloro­meth­yl)-4-(4-chlorophen­yl)-2-oxo-1,2,3,4-tetra­hydro­pyrimidine-5-carboxyl­ate, (III), it is 87.08 (9)° (Bharanidharan *et al.*, 2014 ▸), and in the crystal structure of ethyl 6-methyl-2-oxo-4-(3,4,5-tri­meth­oxy­phen­yl)-1,2,3,4-tetra­hydro­pyrimidine-5-carboxyl­ate, (IV), it is 75.25 (6)° (Novina *et al.*, 2015 ▸). The ethyl acetate group attached to the pyrimidine ring shows an extended conformation [torsion angle C12—C15—O2—C16 = −175.83 (15)°]. The meth­oxy group at C4 is essentially coplanar with the benzene ring [C5—C4—O5—C7 = −1.3 (3)°], whereas the two meth­oxy substituent groups at C2 and C3 deviate significantly from the benzene plane [C3—C2—O3—C9 = 71.6 (2) and C2—C3—O4—C8 = 71.6 (2)°]. The mol­ecular structure is partially stabilized by the C10—H10*A*⋯O1 intra­molecular inter­action (Table 1 ▸), which generates an *S*(6) ring motif.

## Supra­molecular features   

**Figure 2:**
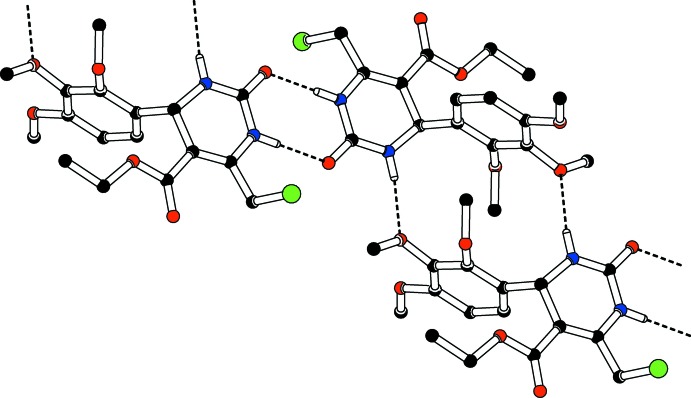
Partial crystal packing diagram for the title compound, showing the 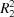
(8) and 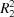
(14) ring motifs. Hydrogen bonds are shown as dashed lines.

**Figure 3:**
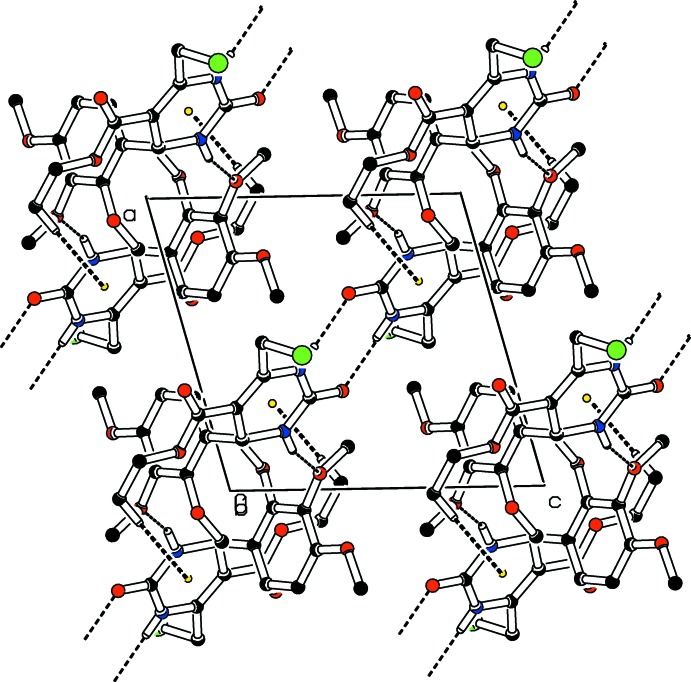
Part of the crystal packing of the title compound, showing C—H⋯π inter­actions and N—H⋯O hydrogen bonds as dashed lines.

In the crystal, both N—H groups participate in inter­molecular hydrogen-bonding associations (Table 1 ▸) giving centrosymmetric cyclic motifs [graph sets 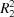
(8) and 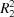
(14)], resulting in ribbons parallel to [111] (Fig. 2 ▸). The packing (Fig. 3 ▸) also features weak C—H⋯π inter­actions between the methyl H atoms of the ethyl groups and the pyrimidine rings of inversion-related mol­ecules.

## Synthesis and crystallization   

To an ethano­lic solution of ethyl 4-chloro­aceto acetate (2 ml, 0.012 mol), 2,3,4-trimeth­oxy benzaldehyde (2.4 g, 0.012 mol), and urea (2.25 g, 0.037 mol) were added followed by CeCl_3_·7H_2_O (931 mg). The reaction mixture was taken in a round-bottom flask and refluxed for 2 h. Then the reaction mixture was cooled and poured into crushed ice taken in a beaker with constant stirring. The solid separated out was filtered, washed with ice-cold water and then recrystallized from hot ethanol to afford the product [yield: 92%; m.p. 425–427 K] as X-ray quality crystals.

## Refinement   

Crystal data, data collection and structure refinement details are summarized in Table 2 ▸. H atoms were placed in geometrically idealized positions and refined as riding on their parent atoms with C—H distances fixed in the range 0.93–0.98 Å and N—H = 0.86 Å with *U*_iso_(H) = 1.5*U*_eq_(CH_3_) and 1.2*U*_eq_(CH_2_,CH, NH).

## Supplementary Material

Crystal structure: contains datablock(s) I. DOI: 10.1107/S2056989015011688/lh5770sup1.cif

Structure factors: contains datablock(s) I. DOI: 10.1107/S2056989015011688/lh5770Isup2.hkl

Supporting information file. DOI: 10.1107/S2056989015011688/lh5770Isup3.cml

CCDC reference: 1407186

Additional supporting information:  crystallographic information; 3D view; checkCIF report

## Figures and Tables

**Table 1:** Hydrogen-bond geometry (, ) *Cg* is the centroid of the N1/C11C13/N2/C14 pyrimidine ring.

*D*H*A*	*D*H	H*A*	*D* *A*	*D*H*A*
N1H1*N*O6^i^	0.86	1.95	2.812(2)	178
N2H2*N*O4^ii^	0.86	2.37	3.160(2)	153
C17H17*C* *Cg* ^iii^	0.96	2.83	3.676(4)	147
C10H10*A*O1	0.97	2.14	2.864(3)	131

Symmetry codes: (i) 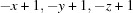; (ii) 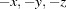; (iii) 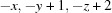.

**Table 2:** Experimental details

Crystal data	
Chemical formula	C_17_H_21_ClN_2_O_6_
*M*_r_	384.81
Crystal system, space group	Triclinic, *P* 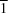
Temperature (K)	293
*a*, *b*, *c* ()	9.479(5), 10.080(5), 10.320(5)
, , ()	108.552(5), 102.886(5), 94.406(5)
*V* (^3^)	899.5(8)
*Z*	2
Radiation type	Mo *K*
(mm^1^)	0.25
Crystal size (mm)	0.20 0.15 0.10

Data collection	
Diffractometer	Bruker Kappa APEXII CCD
Absorption correction	Multi-scan (*SADABS*; Bruker, 2008 ▸)
*T*_min_, *T*_max_	0.952, 0.976
No. of measured, independent and observed [*I* > 2(*I*)] reflections	12878, 3737, 3025
*R*_int_	0.025
(sin /)_max_ (^1^)	0.631

Refinement	
*R*[*F* ^2^ > 2(*F* ^2^)], *wR*(*F* ^2^), *S*	0.041, 0.121, 1.04
No. of reflections	3737
No. of parameters	239
H-atom treatment	H-atom parameters constrained
_max_, _min_ (e ^3^)	0.25, 0.28

Computer programs: *APEX2*, *SAINT* and *XPREP* (Bruker, 2008 ▸), *SIR92* (Altomare *et al.*, 1993 ▸), *SHELXL97* (Sheldrick, 2008 ▸), *ORTEP-3 for Windows* (Farrugia, 2012 ▸) and *PLATON* (Spek, 2009 ▸).

## supplementary crystallographic information

### Crystal data

**Table:**

C_17_H_21_ClN_2_O_6_	*Z* = 2
*M**_r_* = 384.81	*F*(000) = 404
Triclinic, *P*1	*D*_x_ = 1.421 Mg m^−^^3^
Hall symbol: -P 1	Mo *K*α radiation, λ = 0.71073 Å
*a* = 9.479 (5) Å	Cell parameters from 3737 reflections
*b* = 10.080 (5) Å	θ = 1.0–26.6°
*c* = 10.320 (5) Å	µ = 0.25 mm^−^^1^
α = 108.552 (5)°	*T* = 293 K
β = 102.886 (5)°	Block, colourless
γ = 94.406 (5)°	0.20 × 0.15 × 0.10 mm
*V* = 899.5 (8) Å^3^	

### Data collection

**Table:**

Bruker Kappa APEXII CCD diffractometer	3737 independent reflections
Radiation source: fine-focus sealed tube	3025 reflections with *I* > 2σ(*I*)
Graphite monochromator	*R*_int_ = 0.025
ω and φ scan	θ_max_ = 26.6°, θ_min_ = 2.2°
Absorption correction: multi-scan (*SADABS*; Bruker, 2008)	*h* = −11→11
*T*_min_ = 0.952, *T*_max_ = 0.976	*k* = −12→12
12878 measured reflections	*l* = −12→13

### Refinement

**Table:**

Refinement on *F*^2^	Primary atom site location: structure-invariant direct methods
Least-squares matrix: full	Secondary atom site location: difference Fourier map
*R*[*F*^2^ > 2σ(*F*^2^)] = 0.041	Hydrogen site location: inferred from neighbouring sites
*wR*(*F*^2^) = 0.121	H-atom parameters constrained
*S* = 1.04	*w* = 1/[σ^2^(*F*_o_^2^) + (0.0599*P*)^2^ + 0.3029*P*] where *P* = (*F*_o_^2^ + 2*F*_c_^2^)/3
3737 reflections	(Δ/σ)_max_ < 0.001
239 parameters	Δρ_max_ = 0.25 e Å^−^^3^
0 restraints	Δρ_min_ = −0.28 e Å^−^^3^

### Special details

**Table:**

Geometry. All e.s.d.'s (except the e.s.d. in the dihedral angle between two l.s. planes) are estimated using the full covariance matrix. The cell e.s.d.'s are taken into account individually in the estimation of e.s.d.'s in distances, angles and torsion angles; correlations between e.s.d.'s in cell parameters are only used when they are defined by crystal symmetry. An approximate (isotropic) treatment of cell e.s.d.'s is used for estimating e.s.d.'s involving l.s. planes.
Refinement. Refinement of *F*^2^ against ALL reflections. The weighted *R*-factor *wR* and goodness of fit *S* are based on *F*^2^, conventional *R*-factors *R* are based on *F*, with *F* set to zero for negative *F*^2^. The threshold expression of *F*^2^ > σ(*F*^2^) is used only for calculating *R*-factors(gt) *etc*. and is not relevant to the choice of reflections for refinement. *R*-factors based on *F*^2^ are statistically about twice as large as those based on *F*, and *R*- factors based on ALL data will be even larger.

### Fractional atomic coordinates and isotropic or equivalent isotropic displacement parameters (Å^2^)

**Table:**

	*x*	*y*	*z*	*U*_iso_*/*U*_eq_	
C7	0.3380 (3)	−0.2172 (2)	−0.3248 (3)	0.0695 (6)	
H7A	0.3999	−0.1501	−0.3444	0.104*	
H7B	0.3213	−0.3077	−0.3989	0.104*	
H7C	0.3848	−0.2260	−0.2360	0.104*	
C4	0.2052 (2)	−0.03983 (17)	−0.22112 (17)	0.0417 (4)	
C5	0.33170 (19)	0.04788 (18)	−0.12747 (18)	0.0428 (4)	
H5	0.4230	0.0206	−0.1293	0.051*	
C6	0.32077 (18)	0.17612 (18)	−0.03140 (17)	0.0404 (4)	
H6	0.4058	0.2338	0.0317	0.049*	
C1	0.18792 (17)	0.22104 (16)	−0.02627 (16)	0.0353 (3)	
C2	0.06034 (17)	0.13322 (17)	−0.12190 (17)	0.0375 (4)	
C3	0.06932 (19)	0.00217 (17)	−0.21729 (17)	0.0409 (4)	
C8	−0.1441 (3)	−0.0596 (3)	−0.4122 (2)	0.0685 (6)	
H8A	−0.1575	0.0377	−0.3781	0.103*	
H8B	−0.2377	−0.1197	−0.4467	0.103*	
H8C	−0.0969	−0.0740	−0.4877	0.103*	
C9	−0.1707 (3)	0.1099 (3)	−0.0726 (3)	0.0690 (6)	
H9A	−0.1769	0.0092	−0.1149	0.104*	
H9B	−0.2658	0.1358	−0.0970	0.104*	
H9C	−0.1365	0.1373	0.0285	0.104*	
C13	0.17802 (17)	0.35901 (16)	0.08517 (16)	0.0360 (3)	
H13	0.0787	0.3807	0.0601	0.043*	
C12	0.28624 (17)	0.48334 (16)	0.09400 (16)	0.0357 (3)	
C11	0.39561 (18)	0.54845 (16)	0.21121 (17)	0.0376 (4)	
C14	0.31732 (19)	0.40206 (18)	0.33668 (17)	0.0417 (4)	
C10	0.5076 (2)	0.67371 (18)	0.23650 (19)	0.0451 (4)	
H10A	0.5094	0.6851	0.1470	0.054*	
H10B	0.6041	0.6582	0.2793	0.054*	
C15	0.26590 (19)	0.52688 (17)	−0.03168 (17)	0.0391 (4)	
C16	0.1095 (2)	0.4838 (2)	−0.2602 (2)	0.0548 (5)	
H16A	0.1940	0.4739	−0.2989	0.066*	
H16B	0.0869	0.5787	−0.2469	0.066*	
C17	−0.0182 (3)	0.3764 (3)	−0.3576 (2)	0.0816 (8)	
H17A	0.0067	0.2832	−0.3725	0.122*	
H17B	−0.0438	0.3916	−0.4466	0.122*	
H17C	−0.1001	0.3852	−0.3166	0.122*	
N2	0.20306 (15)	0.34077 (15)	0.22426 (14)	0.0414 (3)	
H2N	0.1367	0.2844	0.2341	0.050*	
N1	0.41333 (16)	0.50454 (16)	0.32654 (15)	0.0478 (4)	
H1N	0.4894	0.5439	0.3964	0.057*	
O5	0.20239 (16)	−0.16966 (14)	−0.31766 (15)	0.0586 (4)	
O4	−0.05528 (14)	−0.09329 (13)	−0.29991 (14)	0.0536 (4)	
O3	−0.07186 (13)	0.17960 (13)	−0.12314 (14)	0.0471 (3)	
O1	0.34931 (16)	0.60863 (16)	−0.05290 (15)	0.0615 (4)	
O2	0.13972 (14)	0.45913 (14)	−0.12663 (13)	0.0495 (3)	
O6	0.33671 (15)	0.37184 (15)	0.44510 (13)	0.0595 (4)	
Cl	0.46401 (7)	0.83004 (5)	0.35072 (6)	0.06618 (19)	

### Atomic displacement parameters (Å^2^)

**Table:**

	*U*^11^	*U*^22^	*U*^33^	*U*^12^	*U*^13^	*U*^23^
C7	0.0724 (15)	0.0561 (13)	0.0664 (14)	0.0167 (11)	0.0202 (11)	−0.0003 (10)
C4	0.0495 (10)	0.0351 (8)	0.0351 (8)	0.0004 (7)	0.0089 (7)	0.0082 (7)
C5	0.0384 (9)	0.0438 (9)	0.0440 (9)	0.0025 (7)	0.0120 (7)	0.0123 (7)
C6	0.0355 (8)	0.0396 (8)	0.0382 (8)	−0.0058 (7)	0.0033 (6)	0.0097 (7)
C1	0.0371 (8)	0.0317 (7)	0.0324 (7)	−0.0034 (6)	0.0034 (6)	0.0106 (6)
C2	0.0355 (8)	0.0346 (8)	0.0371 (8)	−0.0010 (6)	0.0016 (6)	0.0119 (6)
C3	0.0411 (9)	0.0340 (8)	0.0369 (8)	−0.0066 (7)	−0.0003 (7)	0.0082 (7)
C8	0.0629 (13)	0.0710 (14)	0.0475 (11)	−0.0165 (11)	−0.0123 (9)	0.0127 (10)
C9	0.0669 (14)	0.0783 (15)	0.0892 (17)	0.0250 (12)	0.0395 (13)	0.0495 (14)
C13	0.0343 (8)	0.0352 (8)	0.0316 (7)	−0.0030 (6)	0.0020 (6)	0.0087 (6)
C12	0.0380 (8)	0.0294 (7)	0.0348 (8)	0.0013 (6)	0.0059 (6)	0.0078 (6)
C11	0.0393 (9)	0.0320 (8)	0.0371 (8)	−0.0010 (6)	0.0078 (7)	0.0089 (6)
C14	0.0439 (9)	0.0385 (8)	0.0351 (8)	−0.0059 (7)	0.0025 (7)	0.0107 (7)
C10	0.0469 (10)	0.0373 (9)	0.0426 (9)	−0.0075 (7)	0.0076 (7)	0.0082 (7)
C15	0.0424 (9)	0.0337 (8)	0.0384 (8)	0.0052 (7)	0.0088 (7)	0.0100 (7)
C16	0.0629 (12)	0.0611 (12)	0.0402 (9)	0.0124 (10)	0.0058 (8)	0.0220 (9)
C17	0.0997 (19)	0.0735 (16)	0.0466 (12)	0.0001 (14)	−0.0117 (12)	0.0114 (11)
N2	0.0422 (8)	0.0407 (7)	0.0328 (7)	−0.0115 (6)	0.0035 (6)	0.0096 (6)
N1	0.0463 (8)	0.0482 (8)	0.0369 (7)	−0.0168 (7)	−0.0066 (6)	0.0160 (6)
O5	0.0615 (9)	0.0436 (7)	0.0531 (8)	0.0061 (6)	0.0102 (6)	−0.0030 (6)
O4	0.0483 (7)	0.0383 (6)	0.0535 (7)	−0.0110 (5)	−0.0075 (6)	0.0066 (6)
O3	0.0359 (6)	0.0398 (6)	0.0581 (8)	−0.0001 (5)	0.0015 (5)	0.0154 (6)
O1	0.0604 (9)	0.0675 (9)	0.0557 (8)	−0.0128 (7)	0.0036 (6)	0.0334 (7)
O2	0.0519 (7)	0.0511 (7)	0.0386 (6)	−0.0040 (6)	−0.0016 (5)	0.0180 (5)
O6	0.0628 (9)	0.0630 (9)	0.0412 (7)	−0.0235 (7)	−0.0084 (6)	0.0250 (6)
Cl	0.0771 (4)	0.0375 (3)	0.0716 (4)	0.0020 (2)	0.0145 (3)	0.0072 (2)

### Geometric parameters (Å, º)

**Table:**

C7—O5	1.415 (3)	C13—N2	1.474 (2)
C7—H7A	0.9600	C13—C12	1.523 (2)
C7—H7B	0.9600	C13—H13	0.9800
C7—H7C	0.9600	C12—C11	1.341 (2)
C4—O5	1.364 (2)	C12—C15	1.475 (2)
C4—C5	1.389 (2)	C11—N1	1.378 (2)
C4—C3	1.391 (3)	C11—C10	1.500 (2)
C5—C6	1.384 (3)	C14—O6	1.230 (2)
C5—H5	0.9300	C14—N2	1.335 (2)
C6—C1	1.377 (3)	C14—N1	1.369 (2)
C6—H6	0.9300	C10—Cl	1.783 (2)
C1—C2	1.403 (2)	C10—H10A	0.9700
C1—C13	1.522 (2)	C10—H10B	0.9700
C2—O3	1.370 (2)	C15—O1	1.202 (2)
C2—C3	1.396 (2)	C15—O2	1.335 (2)
C3—O4	1.3791 (19)	C16—O2	1.448 (2)
C8—O4	1.424 (3)	C16—C17	1.487 (3)
C8—H8A	0.9600	C16—H16A	0.9700
C8—H8B	0.9600	C16—H16B	0.9700
C8—H8C	0.9600	C17—H17A	0.9600
C9—O3	1.412 (2)	C17—H17B	0.9600
C9—H9A	0.9600	C17—H17C	0.9600
C9—H9B	0.9600	N2—H2N	0.8600
C9—H9C	0.9600	N1—H1N	0.8600
			
O5—C7—H7A	109.5	C12—C13—H13	108.2
O5—C7—H7B	109.5	C11—C12—C15	122.12 (15)
H7A—C7—H7B	109.5	C11—C12—C13	120.78 (14)
O5—C7—H7C	109.5	C15—C12—C13	117.09 (13)
H7A—C7—H7C	109.5	C12—C11—N1	120.98 (14)
H7B—C7—H7C	109.5	C12—C11—C10	126.74 (15)
O5—C4—C5	124.55 (17)	N1—C11—C10	112.26 (14)
O5—C4—C3	115.73 (15)	O6—C14—N2	123.26 (15)
C5—C4—C3	119.71 (16)	O6—C14—N1	120.67 (14)
C6—C5—C4	119.45 (17)	N2—C14—N1	116.06 (15)
C6—C5—H5	120.3	C11—C10—Cl	109.90 (13)
C4—C5—H5	120.3	C11—C10—H10A	109.7
C1—C6—C5	122.07 (15)	Cl—C10—H10A	109.7
C1—C6—H6	119.0	C11—C10—H10B	109.7
C5—C6—H6	119.0	Cl—C10—H10B	109.7
C6—C1—C2	118.47 (15)	H10A—C10—H10B	108.2
C6—C1—C13	121.07 (13)	O1—C15—O2	122.11 (16)
C2—C1—C13	120.39 (15)	O1—C15—C12	127.08 (15)
O3—C2—C3	120.68 (14)	O2—C15—C12	110.79 (14)
O3—C2—C1	119.18 (15)	O2—C16—C17	107.07 (17)
C3—C2—C1	120.11 (16)	O2—C16—H16A	110.3
O4—C3—C4	118.51 (15)	C17—C16—H16A	110.3
O4—C3—C2	121.07 (16)	O2—C16—H16B	110.3
C4—C3—C2	120.16 (14)	C17—C16—H16B	110.3
O4—C8—H8A	109.5	H16A—C16—H16B	108.6
O4—C8—H8B	109.5	C16—C17—H17A	109.5
H8A—C8—H8B	109.5	C16—C17—H17B	109.5
O4—C8—H8C	109.5	H17A—C17—H17B	109.5
H8A—C8—H8C	109.5	C16—C17—H17C	109.5
H8B—C8—H8C	109.5	H17A—C17—H17C	109.5
O3—C9—H9A	109.5	H17B—C17—H17C	109.5
O3—C9—H9B	109.5	C14—N2—C13	127.23 (14)
H9A—C9—H9B	109.5	C14—N2—H2N	116.4
O3—C9—H9C	109.5	C13—N2—H2N	116.4
H9A—C9—H9C	109.5	C14—N1—C11	124.02 (14)
H9B—C9—H9C	109.5	C14—N1—H1N	118.0
N2—C13—C1	109.46 (13)	C11—N1—H1N	118.0
N2—C13—C12	109.91 (12)	C4—O5—C7	117.72 (15)
C1—C13—C12	112.61 (13)	C3—O4—C8	117.15 (15)
N2—C13—H13	108.2	C2—O3—C9	116.65 (15)
C1—C13—H13	108.2	C15—O2—C16	117.05 (14)
			
O5—C4—C5—C6	−178.30 (16)	C13—C12—C11—N1	0.2 (3)
C3—C4—C5—C6	0.0 (3)	C15—C12—C11—C10	2.3 (3)
C4—C5—C6—C1	−0.7 (3)	C13—C12—C11—C10	−178.40 (16)
C5—C6—C1—C2	0.0 (2)	C12—C11—C10—Cl	103.68 (19)
C5—C6—C1—C13	176.89 (15)	N1—C11—C10—Cl	−75.01 (18)
C6—C1—C2—O3	−176.62 (14)	C11—C12—C15—O1	9.9 (3)
C13—C1—C2—O3	6.5 (2)	C13—C12—C15—O1	−169.41 (18)
C6—C1—C2—C3	1.4 (2)	C11—C12—C15—O2	−171.87 (16)
C13—C1—C2—C3	−175.49 (14)	C13—C12—C15—O2	8.8 (2)
O5—C4—C3—O4	5.7 (2)	O6—C14—N2—C13	−173.33 (17)
C5—C4—C3—O4	−172.75 (15)	N1—C14—N2—C13	7.7 (3)
O5—C4—C3—C2	179.86 (15)	C1—C13—N2—C14	112.29 (19)
C5—C4—C3—C2	1.4 (3)	C12—C13—N2—C14	−11.9 (2)
O3—C2—C3—O4	−10.1 (2)	O6—C14—N1—C11	−177.08 (18)
C1—C2—C3—O4	171.86 (15)	N2—C14—N1—C11	1.9 (3)
O3—C2—C3—C4	175.89 (15)	C12—C11—N1—C14	−5.6 (3)
C1—C2—C3—C4	−2.1 (2)	C10—C11—N1—C14	173.14 (17)
C6—C1—C13—N2	−72.80 (18)	C5—C4—O5—C7	−1.3 (3)
C2—C1—C13—N2	104.02 (16)	C3—C4—O5—C7	−179.67 (18)
C6—C1—C13—C12	49.8 (2)	C4—C3—O4—C8	−114.3 (2)
C2—C1—C13—C12	−133.40 (15)	C2—C3—O4—C8	71.6 (2)
N2—C13—C12—C11	7.4 (2)	C3—C2—O3—C9	71.6 (2)
C1—C13—C12—C11	−114.95 (17)	C1—C2—O3—C9	−110.4 (2)
N2—C13—C12—C15	−173.26 (14)	O1—C15—O2—C16	2.5 (3)
C1—C13—C12—C15	64.41 (19)	C12—C15—O2—C16	−175.83 (15)
C15—C12—C11—N1	−179.15 (15)	C17—C16—O2—C15	168.35 (19)

### Hydrogen-bond geometry (Å, º)

Cg is the centroid of the N1/C11–C13/N2/C14 pyrimidine ring.

**Table:**

*D*—H···*A*	*D*—H	H···*A*	*D*···*A*	*D*—H···*A*
N1—H1*N*···O6^i^	0.86	1.95	2.812 (2)	178
N2—H2*N*···O4^ii^	0.86	2.37	3.160 (2)	153
C17—H17*C*···*Cg*^iii^	0.96	2.83	3.676 (4)	147
C10—H10*A*···O1	0.97	2.14	2.864 (3)	131

Symmetry codes: (i) −*x*+1, −*y*+1, −*z*+1; (ii) −*x*, −*y*, −*z*; (iii) −*x*, −*y*+1, −*z*+2.
